# Epithelioid hemangioendothelioma of the right atrium invaded the superior vena cava: case report and review of literature

**DOI:** 10.1007/s10554-020-01963-w

**Published:** 2020-08-18

**Authors:** Wenpeng Huang, Liming Li, Jianbo Gao, Jian-Bo Gao

**Affiliations:** Department of Radiology, The First Affiliated Hospital of Zhengzhou University, No.1 Eastern Jianshe Road, Zhengzhou, 450052 China

**Keywords:** Heart neoplasms, Epithelioid hemangioendothelioma, Tomography, X-ray computed, Magnetic resonance imaging

## Abstract

Epithelioid hemangioendothelioma (EHE) is a rare hemangioma that can occur anywhere in the body. It occurs most commonly in the liver and lungs, rarely from the heart, and the etiology or risk factors are unclear. So far, timely detection and radical resection is a more acceptable treatment. Reviewing the literature, few cases of cardiac EHE have been reported. We present a rare case of EHE of the right atrium invaded the superior vena cava.

## Introduction

Epithelioid hemangioendothelioma (EHE) is a rare low-grade malignant angiogenic tumor with local invasiveness and metastatic potential. EHE is a rare low-grade malignant angiogenic tumor with local invasiveness and metastatic potential. Clinically, EHE can occur simultaneously or sequentially in many parts, such as lung, liver, bone, soft tissue and other organs, and there is no obvious specificity in clinical symptoms. There are few case reports of EHE in the literature. We report a unique case of EHE of the right atrium invaded the superior vena cava and review of the literature.

## Case report

A 54-year-old man had intermittent chest tightness for 49 years. There was no obvious inducement of facial and eyelid edema 2 months ago, and it was aggravated when he got up in the morning. However, physical examination found that the patient did not have dyspnea, hemoptysis, cyanosis, limb edema, sensory abnormalities and other symptoms. There is no previous history of surgery or other diseases. Laboratory examination showed that hemoglobin 115.0 g/L, erythrocyte 3.63 × 10^12^, 24-h urine protein and tumor markers were not abnormal.

**Fig. 1:**
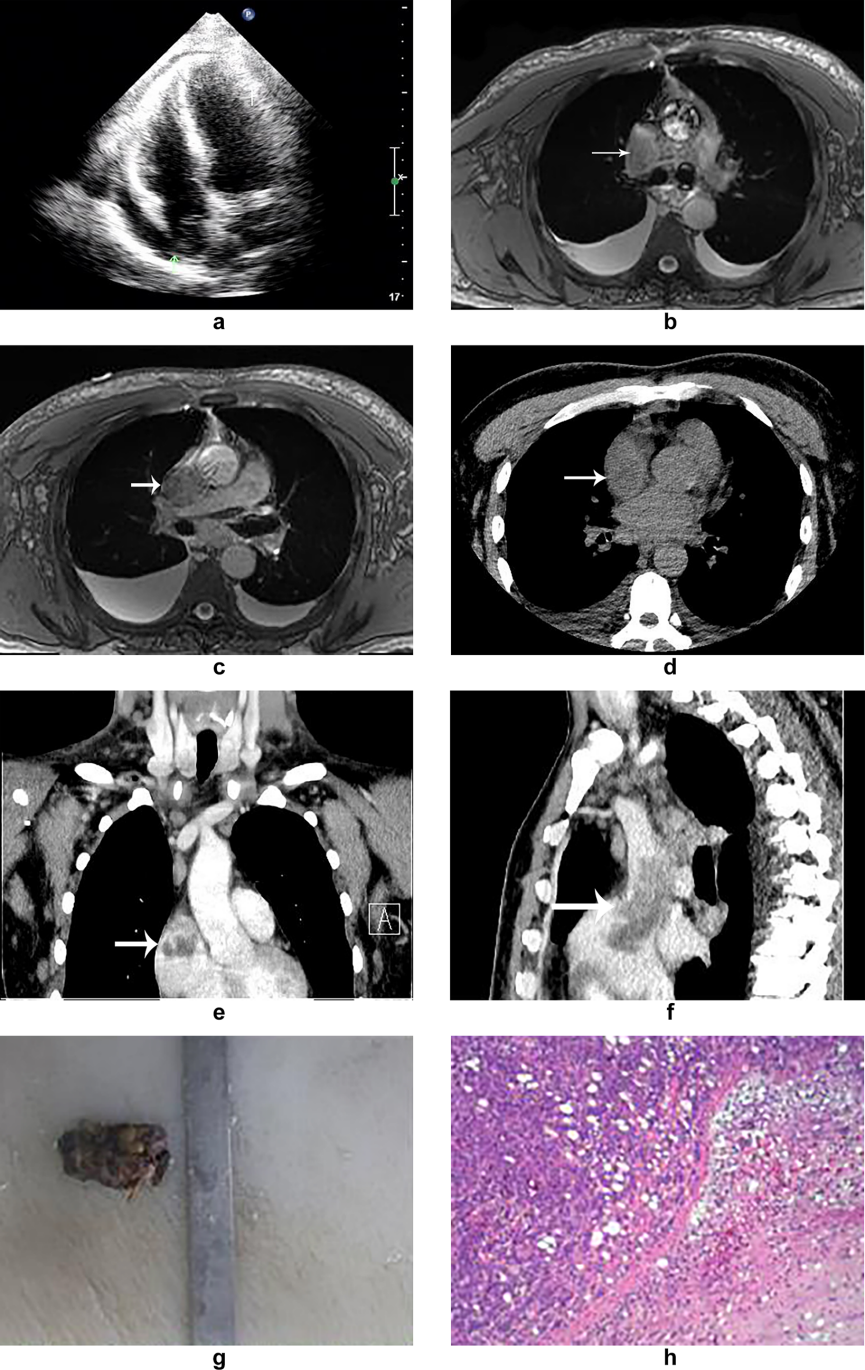
**a** Echocardiography showed that hypoechoic attachment to the heart wall could be found in the right atrium near the entrance of the superior vena cava (green arrow). **b** Cardiac magnetic resonance trufi sequence showed a mass of low signal intensity in the right atrium (white arrow). **c** The delayed contrast-enhanced sequence of cardiac magnetic resonance imaging showed that the lesions were slightly enhanced in mass (white arrow). **d** CT plain scan showed patchy low-density shadow in the right atrium and superior vena cava (white arrow). **e** Re-formatted multiple planar reformation image showing inhomogeneous and moderate enhancement of the low-density shadow in the right atrium (white arrow). **f** Re-formatted multiple planar reformation image showing the lesions invading the superior vena cava (white arrow). **g** The size of the tumor was about 6.5 cm × 3.0 cm × 2.5 cm and the capsule was intact. **h** The tumor cells in the tissue were epithelioid, the atypia was not obvious, and mitosis was rare. Vacuoles of different sizes were seen in the eosinophilic cytoplasm, forming a vascular cavity-like structure

After admission, the cardiac function of the patient was examined. First, echocardiography showed that the right atrium near the entrance of the superior vena cava could be hypoechoically attached to the heart wall, with an area of about 22 mm × 16 mm (Fig. 1a). The fluid dark area was detected in the pericardium. Ultrasound doctors considered the formation of emboli extending from the right atrium to the entrance of the inferior vena cava. Subsequently, the patient underwent cardiac magnetic resonance imaging, showing a massive low-signal shadow in the right atrium on the trufi sequence (Fig. 1b). The right atrium was small and the right ventricle was large, so delayed contrast-enhanced scan was performed 2 h later. After intravenous injection of contrast medium, the lesions in the superior vena cava near the heart and adjacent right atrium showed mild enhancement (Fig. 1c), multiple nodular mild to moderate enhancement foci could be seen in the mediastinum, and a small or moderate amount of effusion could be seen in the pericardial cavity. Dr. MRI considered the mass in the proximal end of the superior vena cava and adjacent right atrium. Further CT examination showed patchy low density shadow in right atrium and superior vena cava (Fig. 1d), inhomogeneous and moderate enhancement on enhanced scan (Fig. 1e–f), multiple enlarged lymph nodes and spotted calcification in mediastinum and bilateral axillary fossa, localized thickening of pericardium and fluid density in pericardium and bilateral chest. In addition, PET-CT examination showed that the density of soft tissue was concentrated in the right atrium and superior vena cava, SUV_max_ was about 10.7, the length of the lesion was about 6.7 cm, multiple enlarged lymph nodes were seen in mediastinum, bilateral hilum and bilateral armpit, some of them were slightly concentrated, SUV_max_ was about 3.3, and the size of larger patients was about 1.3 cm × 1.5 cm.

The patient underwent surgical resection. During the operation, it was found that the left and right brachial trunk vein and superior vena cava were enlarged, the tumor was located in the right atrium, the size was about 6.5 cm × 3.0 cm × 2.5 cm (Fig. 1g), and the capsule was intact. The tumor section is gray–yellow–gray–red soft, locally cystic, and the boundary with the heart tissue is still clear. the tumor pedicle was located in the atrial septum, part of the tumor grows upward to the vena cava, attaches part of the thrombus, and completely blocks the blood flow of the superior vena cava. After the tumor was sent for histopathological examination, it could be seen that the tumor tissue was composed of scattered heterosexual epithelial cells and spindle cells, the mitosis of the cell core was rare, and the vascular lumen-like structure could be seen in some eosinophilic cytoplasm (Fig. 1h). Immunohistochemistry showed CK(−), CK7(−), CEA(−), TTF-1(−), CK5/6(−), CR(−), HBME-1(−), D2-40(−), WT-1(−), P63(−), CD31(+), CD34(−), ERG(+), Ki-67(12%+), FLI-1(+), TFE-3(−), F8(+)。. The patients were followed up every 3 months after operation. 15 months after operation, the patients were in good condition and there were no obvious complications.

## Discussion

Primary cardiac tumors are very rare, about 20–30% are malignant tumors [1]. Epithelioid hemangioendothelioma (EHE) is a rare hemangioma originating from vascular endothelial cells orpre-endothelial cells, first described by Weiss and Enzinger in 1982 [2]. Its biological behavior is between benign hemangioma and malignant angiosarcoma, and it has local invasiveness and metastatic potential. Metastases (lung, lymph nodes, liver, bones, retroperitoneum, soft tissues) and death can occur in about 25 and 15% of EHE, respectively [3].In the new WHO classification in 2002, this tumor was classified as a malignant vascular tumor in soft tissue tumors and classified as a low-grade malignant angiogenic tumor [4]. It is reported that EHE originates from almost every organ system, including lung, liver, bone, soft tissue, limbs, spleen and other organs, but rarely occurs in the heart. The cause is not clear and may be related to trauma, radiotherapy and hormone levels [5].

An extensive literature search was performed on PubMed databases using the keyword- Epithelioid hemangioendothelioma and heart. The reference lists of all retrieved studies were scrutinized for additional articles to supplement the search result. All the duplicates articles were excluded. To our knowledge there have been 22 cases of Epithelioid hemangioendothelioma of the heart in the English literature. These are summarized in Table 1. Most of the studies are case reports, and the clinical symptoms of this kind of tumor with unknown etiology have no obvious specificity, which mainly depends on the location and size of the tumor. If it occurs in the heart, it may show respiratory distress, hemoptysis, palpitations, chest pain, or no obvious manifestation. It can occur in any part of the heart, of which the right atrium is the most common site of, The mean age in the reported case reports was 45 years (range 2 months−77 years), there is no significant gender difference.

**Table 1:** Clinical and pathological characteristics and management of cardiac EHE

Sr.no.	Author (year)	Age (year)	Sex	Camber	Size (cm)	Presenting symptoms	Recurrence	Final therapy	Follow up
1	Rosai et al. [9] (1979)	25	M	Left atrium	None	Rheumatic heart disease with mitral valve stenosis	None	Resection	Survived
2	Hayward et al. [10] (1979)	49	F	Mitral valve, PML chorda	NA	Diastolic murmur typical of mitral stenosis	NA	Resection	Survived
3	Kuo et al. [11] (1985)	65	M	Left atrium	None	Recalcitrant pruritus	None	Resection	Survived
4	Singal et al. [12] (1987)	19	F	Left atrium	4 × 4 × 5	Heart murmur	None	Resection	Liver metastasis
5	Montes et al. [13] (1991)	56	F	Right ventricle	8 × 5 × 4	Pulmonary stenosis	None	Resection	Pulmonary metastasis (4 months)
6	Marchiano et al. [14] (1993)	71	F	Left atrium	5.5 × 4.5 × 3.5	Palpitations, dizziness	None	Resection	Buttock metastasis (4 months)
7	Bille et al. [10] (1993)	59	M	Aortic valve	0.5	Cerebral infarction	NA	Resection	NA
8	Biasi et al. [15] (1995)	35	M	Right ventricular	2 × 3.5	Moderate mitral regurgitation	None	Resection	Survived
9	Bisesi et al. [16] (1996)	12	F	Right atrium	None	Hemoptysis	None	Conservative	NA
10	Agaimy et al. [17] (2002)	68	M	Right ventricle	0.8	Incidentally discovered on autopsy	NA	NA	NA
11	Tansel et al. [18] (2005)	2 mouths	F	Left atrium	None	Respiratory distress	None	Resection	Survived
12	Kitamura et al. [10] (2005)	36	F	Right atrium	12 × 11	Cough, lung edema	None	Resection	Survived
13	Val-Bernal et al. [19] (2005)	69	F	Left ventricle	0.4	Incidentally discovered on echocardiogram	None	Resection	Survived
14	Moulai et al. [10] (2006)	53	M	Cardiac mass invading	NA	Incidentally discovered on echocardiogram	None	Chemotherapy	Survived
15	Safirstein et al. [5] (2007)	51	F	Right atrium	5 × 4 × 4	Incidentally discovered on echocardiogram	None	Resection	Survived
16	Lisy et al. [20] (2007)	61	M	Left atrium	4.2	Incidentally discovered on computed tomography	None	Resection	Survived
17	Messias et al. [10] (2008)	21	F	Left atrium	3.9 × 2.7	Chest pain	None	Resection	Survived
18	Sugimoto et al. [10] (2013)	77	F	Right atrium	2.5	Incidentally discovered on echocardiogram	None	Resection	Survived
19	Allain et al. [21] (2014)	35	M	Right atrium	6 × 7 × 11	Rapidly progressive dyspnea	None	Resection	Survived
20	Ellouze et al. [10] (2015)	53	M	Right atrium	2.7 × 2.8	Chest pain and palpitation	None	Resection	Pulmonary metastasis ( 10 months)
21	Patel et al. [22] (2018)	49	M	Right atrium	4 × 4	Syncope	None	Resection	NA
22	Wu et al. [23] (2019)	32	M	Right atrium	4.1 × 5.9	Palpitations, chest tightness and chest pain	None	Resection	Survived

EHE has unique histological, immunohistochemical and molecular characteristics. Histologically, round or polygonal endothelial cells were arranged in nests and cords. The cytoplasm of tumor cells is usually rich in eosinophilic hyaline and the presence of cytoplasmic vacuoles and vesicular nuclei. Immunohistochemistry is likewise useful in contributing to the diagnosis. The vascular nature of EHE is identified by Friend leukemia integration 1 transcription factor (FLI-1), which is a transcription factor, expressed in endothelial cells [6]. CD34 is expressed in more than 90% of vascular tumors, so although it is relatively sensitive, it is not very specific to EHE. In contrast, CD31 is a more specific vascular tumor marker, so some scholars recommend immunohistochemical staining combined with CD31, ERG, FLI-1 as an important index for the diagnosis of EHE. CD31[7], ERG and FLI-1 are all positive in our patient.

The imaging findings of cardiac EHE were not specific. Our patient’s EHE was located in the right atrium, part of the tumor grew upward to the vena cava, CT plain scan showed patchy low density, and enhanced scan showed inhomogeneous and moderate enhancement,Multiple lymph nodes and spotted calcification could be seen in the mediastinum; MRI showed mass iso-T1 and slightly short T2 signal intensity, delayed enhancement showed mild enhancement, and multiple nodular mild to moderate enhancement lesions in the mediastinum. Before operation, the imaging diagnosis was misdiagnosed as thrombus formation in the right atrium and superior vena cava, but in this case, the density of the filling defect was uneven and there was enhancement, and there were no blood vessels in the thrombus, so the possibility of the tumor should be considered. and multiple spots of calcification can be seen in the mediastinum, indicating that the mass invades the mediastinum, but the thrombus will not invade the mediastinum.

At present, surgical treatment is the main treatment of cardiac EHE. Some chemotherapeutic drugs and radiotherapy regimens have been reported in affected patients, but there are no significant therapeutic benefits. Because the sensitivity of the heart to radiation injury, resulting in cardiomyopathy or chronic pericarditis at therapeutic dosages, limits the benefits of radiotherapy。.

The prognosis of cardiac epithelioid hemangioendothelioma is unpredictable. the tumor may stop growing, or it may recur and metastasize. Clinical symptoms, tumor lymphatic vessel spread, distant organ metastasis and peripheral lymph node lesions have all been shown to be associated with poor prognosis [8]. After discharge, patients still need long-term follow-up to monitor for distant metastasis or recurrence. In the review of the literature, postoperative metastasis was reported in 4 cases. Our patients survived disease-free for a long time and were followed up for 15 months without recurrence or metastasis. We cannot rule out the possibility of recurrence of this kind of tumor in the future, because delayed recurrence may occur in EHE after many years.

## Conclusion

In summary,Cardiac EHE is rare and the prognosis and clinical behavior are uncertain. Long-term follow-up of patients after discharge is necessary in order to more accurately evaluation the prognosis of cardiac EHE. We present the case report of the right atrium invaded the superior vena cava. In our patient, was effectively treated with surgery management and in concordance with the available literature.

## Abbreviations

EHE: Epithelioid hemangioendothelioma
CT: Computed tomography
MRI: Magnetic resonance imaging
FLI-1: Friend leukemia integration 1
